# Comparison between mechanical properties of human saphenous vein and umbilical vein

**DOI:** 10.1186/1475-925X-11-59

**Published:** 2012-08-23

**Authors:** Borhan Alhosseini Hamedani, Mahdi Navidbakhsh, Hossein Ahmadi Tafti

**Affiliations:** 1 M. Sc in Biomedical Engineering, Biomechanics Lab, School of Mechanical Engineering, Iran University of Science and Technology, Tehran, Iran; 2 Associated Professor, School of Mechanical Engineering, Iran University of Science and Technology, Tehran, Iran; 3 MD, Professor, cardiothoracic surgery department, Tehran Heart Center, Tehran, Iran

**Keywords:** Umbilical vein, Saphenous vein, Non-linear rheological behavior, Fourth order polynomial, Cauchy stress, Stretch ratio.

## Abstract

**Background:**

As a main cause of mortality in developed countries, Coronary Artery Disease (CAD) is known as silent killer with a considerable cost to be dedicated for its treatment. Coronary Artery Bypass Graft (CABG) is a common remedy for CAD for which different blood vessels are used as a detour. There is a lack of knowledge about mechanical properties of human blood vessels used for CABG, and while these properties have a great impact on long-term patency of a CABG. Thus, studying these properties, especially those of human umbilical veins which have not been considered yet, looks utterly necessary.

**Methods:**

Umbilical vein, as well as human Saphenous vein, are respectively obtained after cesarean and CABG. First, histological tests were performed to investigate different fiber contents of the samples. Having prepared samples carefully, force-displacement results of samples were rendered to real stress–strain measurements and then a fourth-order polynomial was used to prove the non-linear behavior of these two vessels.

**Results:**

Results were analyzed in two directions, i.e. circumferentially and longitudinally, which then were compared with each other. The comparison between stiffness and elasticity of these veins showed that Saphenous vein’s stiffness is much higher than that of umbilical vein and also, it is less stretchable. Furthermore, for both vessels, longitudinal stiffness was higher than that of circumferential and in stark contrast, stretch ratio in circumferential direction came much higher than longitudinal orientation.

**Conclusion:**

Blood pressure is very high in the region of aorta, so there should be a stiff blood vessel in this area and previous investigations showed that stiffer vessels would have a better influence on the flow of bypass. To this end, the current study has made an attempt to compare these two blood vessels’ stiffness, finding that Saphenous vein is stiffer than umbilical vein which is somehow as stiff as rat aortic vessels. As blood vessel’s stiffness is directly related to elastin and mainly collagen content, results showed the lower amount of these two contents in umbilical vein regarding Saphenous vein.

## Background

Coronary Artery Disease is the main cause of mortality in developed countries. In 2006, according to American Heart Association (AHA), more than 80,000,000 American adults experienced at least one type of cardiovascular disease. Among these, Coronary Heart Disease (CHD) had the greatest proportion of mortality with 425,000 among 831,000 (51% of mortality because of cardiovascular disease) [1]. When damaged, the artery should be replaced with another vessel. For vessels larger than 6 mm diameter, artificial replacements provide acceptable results, but small-diameter vessels have the problem of thrombosis and intimal hyperplasia [2]. In coronary occlusion, different arteries and veins like mammary artery, radial artery, and Saphenous Vein (SV) are used as a detour in CABG to provide myocardium demand of oxygen and other nutrients. Because of cyclic high pressure in aorta, it is important to find a stiffer vessel that not only endures this pressure, but also facilitates the coronary and bypass flow. Unfortunately, one third of the SV grafts are occluded after 10 years, whereas mammary shows more inspiring results [3-5].

In 1974 Mc. Donald showed that main arteries have a maximum deformation of 5% in systole [6] and later in 2001, Alderson used an analytical method to demonstrate the efficacy of a stiffer artery in bypass graft. As stiffer artery is used, the pulse wave of graft synchronizes with that of coronary and flow reduction as an asynchrony will be improved. If we assume a beating rate of 60(BPM) for an old man, there are more than 315 million cyclic loads within 10 years for blood vessels and this accentuates the importance of selection of a suitable blood vessel [5].

There are three main methods found in the literature to determine elasticity of blood vessels. The first one is based on pressure distention in the blood vessel in which pressure-artery diameter relationship is used to obtain blood vessel’s compliance and stiffness. Milesi used this method for human SV in normotensive and hypertensive patients who had Coronary Artery Disease (CAD) and showed that hypertensive vessels are stiffer than normotensive vessels [7].Similarly, Carolien, compared coronary artery as well as mammary artery for both human and porcine arteries. She found that although these arteries dimensions are similar, human’s arteries are much stiffer than that of porcine. Additionally, human coronary artery and internal mammary artery were found to possess the same stiffness which may be a strong parameter leading to long-term patency of mammary grafts [8].

Empirically, the most thorough investigation on elastomechanical properties of human SV in the literature belongs to Donovan et al. in 1990, since he studied 45 samples in two orthogonal directions [9]. Miyamotto continued Donovan’s studies by comparing the ultimate strength of SV and bovine treated pericardium as an alternative for carotid endarterectomy [10]. More recently Matthews has compared porcine pulmonary and aorta [11].

In the second method, blood vessel’s elasticity is obtained directly in vivo and because it is a non-invasive measurement, it has considerably been welcomed by researchers. This method is known as Pulse Wave Velocity (PWV) which is based on Moens–Korteweg equation. Here, to determine blood vessel’s elasticity, pressure-diameter relationship and velocity of pulse transfer should be assessed simultaneously [12].

Finally, in the third method, mechanical properties of blood vessels are obtained using tensiometers (or universal testing machine). Large deformation formulation is introduced to be appropriate in stress-stretch relations for soft tissue; nonetheless, some have used engineering stress–strain relationship to model the behavior of blood vessels under tension. For example, Rossman used engineering stress and strain relationships for jugular and lumbar bovine veins [13] and so did Balaz for porcine coronary arteries [14]. Applying large deformation formulation, Teng et al. determined elastomechanical properties of media and adventitia for atherosclerotic human carotid using Piola-Kirchoff stress tensor [15]. Assoul had a comprehensive study on abdominal and thoracic aorta of rat. Effect of frequency and rate of tension was widely inspected in his experiments. In addition, he employed a fourth order polynomial to show the nonlinear behavior of blood vessels [2]. Holzapfel had also an overwhelming analysis on different heart-related blood vessels obtaining their elastomechanical properties with a multilayer assumption [16,17].

Higher amounts of collagen fibers are the cause of greater stiffness in the arteries and veins [18]. Aging is one of the crucial parameters that affects elastomechanical properties of blood vessels in which collagen fibers dwindles with a decrease in Young modulus and ultimate stiffness reduction [19].

Umbilical cord is composed of two arteries and a vein that the vein is employed for bypass surgeries. First plantation with Human Umbilical Vein (HUV) was performed in 1973 in the United States [20]. This vessel was mainly applied for femoropopliteal surgery to supply the blood for thigh and leg. An eleven year survey with the aim of comparison between different conduits in femoropopliteal bypass showed that SV demonstrates better long-term patency versus HUV while the latter shows less failure concerning synthetic conducts like PTFE (Poly Tetra Fluro Ethylen) grafts [21].

On the other hand, from histological point of view, a wide range of existing blood vessels have been investigated. Becoming worthy as an alternative vessel for bypass surgery, many researchers have focused on ultrastructure of HUV [22,23]. Instead of an adventitia layer, this vessel is supported by a mucopolysaccharide-filled layer with sparse amounts of collagen fibers known as Wharton jelly [24]. On average, this vessel comprises of 29.32% collagen fibers and 8.08% elastin [20]. Collagen/Elastin (C/E) ratio is a factor demonstrating the stiffness and elasticity of blood vessel [20]. For SV, higher amounts of these fibers (49.6% and 10% on average, for collagen and elastin respectively) mean a stiffer vessel compared to HUV [18,20,25].On the other hand, HUV is more similar to moderate arteries, while this ratio in SV is evidently the reverse of that in the aortic wall that demonstrates the higher elasticity of aorta than SV [18,20,25].

A research conducted by Li et al. to assess the feasibility of HUV as an alternative for small-caliber vessels revealed that microstructural components of this vessel are similar to that of small-caliber arteries (e.g., brachial, ulnar, radial, right coronary, anterior tibial, and posterior tibial) with moderate amounts of collagen and elastin as well as similar C/E ratio. These strong correlations boosted the possibility of exploiting HUV as a decent vein for bypass surgery [20].

In spite of these valuable studies, there is still a lack of information in the literature about mechanical properties of human SV and also, data for HUV stiffness is utterly sparse. Although autogenous blood vessels possess many advantages, sources are so limited that clinicians usually have to use synthetic blood vessels as substitutes [20]. Seemingly, histological similarity brought together with homogeneity between graft and host artery are two determinants for long-term patency transplantation; so that compliance mismatch between host and transplantation artery is found to be crucial [20].

The aim of current study is to scrutinize the mechanical stiffness and elasticity of two blood vessels; SV that is commonly used to compensate lack of blood flow in myocardium and HUV as a controversial blood vessel for transplantation. Having investigated these properties, we employed a fourth-order polynomial, introduced by Fung [26], so as to determine the stiffness of these vessels and consequently to obtain the instant elasticity of each vessel, more straightforwardly.

## Methods

The followed procedure was approved by ethical committee in Tehran Heart Center based on 2008 Declaration of Helsinki. So, the samples were collected by the permission of patients and pregnants. Before performing any test with human samples, three plastic tubes were examined in both circumferential and longitudinal direction applying Universal Testing Machine (UTM) (Zwick/Roell-HCR 25/400, Germany) to investigate the quality of tests. Acquisition frequency was set to 200 points-per-second and the results showed an excellent correlation with precise available results compared to those of company. Thus, human samples were prepared for tests.

### Preparation of blood vessel

As soon as the bypass operation finished, human thigh SV was prepared from Tehran Heart Center, Tehran, Iran and also after cesarean, HUV was prepared and stripped from umbilical cord in anatomy faculty of Tehran University, Tehran, Iran. SV, after bypass operation, was stored in patient’s heparinized blood [13] while HUV was stored in PBS [2]; these solutions provide blood vessels nutrients preventing dehydration. Their containers were transferred to a Lab in the ice and then they were refrigerated.

To investigate microstructural components of the tested vessels, in addition to mechanical tests, sections of harvested samples were fixed in 10% buffered formalin, and having embedded in paraffin, they were sectioned in 4 μm for histology. Different staining methods were performed to investigate microstructure of these veins; firstly, Haemotoxylin and Eosin (HE) staining approach recruited that dyes all cells nuclei blue with various red tones for other structural elements. Secondly, to dye collagen fibers, Van Gison’s staining was used and finally, Weigert’s resorcin-fuchsin technique, demonstrating the content of elastin fibers, was utilized. Microstructural components were observed under a ZEISS (Munich, Germany) confocal microscope and images were captured using a digital camera.

**Figure 1:**
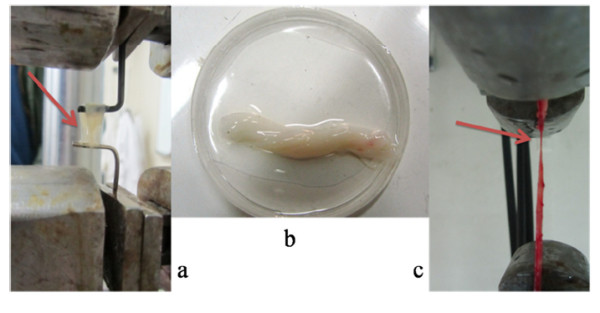
a) tearing the HUV in circumferential direction, b) HUV samples in normal saline solution c: tear of SV in transversal direction.

To test samples mechanically, having prepared the UTM, the vessels were washed with special care using 0.9% normal saline; so that it avoids damaging endothelial layer. Then, specimens were placed in a Petri dish filled with normal saline solution. Although studies have shown that blood vessel cells function up to 24 h [7], all the experiments were conducted within a maximum of 4 hours after surgery. Vein thickness was measured with micrometer at the section of the vessel and the average diameter of blood vessel was obtained with an open section of it applying caliper. 16 SV and 10 HUV were prepared for Experiments, but only 5 samples for SV and 3 samples for HUV were truly tested. Other samples either tore near the jaws which is known as a sign of stress concentration or slipped due to the loose connection between jaw and sample. Figure 1 depicts the samples and their rupture in both directions with primary data for both veins prepared in Table 1.

**Table 1:** Primary data of these vessels

**Sample**	**Sex**	**Age**	**Vessel perimeter (mm)**	**Wall thickness (mm)**
SV 1	Male	64	13	0.5
SV 2	Male	58	13.7	0.51
SV 3	Male	68	12	0.56
SV 4	Female	57	12.3	0.53
SV 5	Female	59	11	0.49
UV 1	-	-	13.1	1.72
UV 2	-	-	14.2	1.56
UV 3	-	-	11.3	1.1

### Primary measurements

To avoid samples slipping in longitudinal tension tests, both ends of the vessels were dried first with paper tissues and then with sandpaper to tightly fasten the vessels to the jaws. Length of the specimen under the force of 0.1 (N) was assumed as primary length in both orientations to make sure that the samples are not free and Assoul’s procedure was applied as a reference for conducting tests [2]. In circumferential cases, the samples were placed between two hooks and the stretch ratio was determined as:

(1) λ=LL0

Where λ represents stretch ratio, and L (mm) and L_0_ (mm) are primary and instant length, respectively. During the test, 0.9% normal saline was sprayed in order to prevent samples dehydration. To relieve the residual stresses, the specimens were stretched to λ = 1.1 for 10 cycles with 1(%/s) rate of elongation; this part of the test lasted for 200 seconds. Immediately after this step, specimens were stretched again with the rate of 1(%/s) until final rupture and force-displacement data were recorded. Table 2 illustrates the maximum load and corresponding displacement of the samples in both directions.

**Table 2:** Load, displacement and initial length of the samples

**Specimen**	**Length (mm)**	**Diameter (mm)**	**Maximum load in longitudinal tension (N)**	**Displacement in maximum longitudinal load (mm)**	**Sample’s width (mm)**	**Maximum load in circumferential tension (N)**	**Displacement in maximum circumferential load (mm)**
SV 1	78	4.13	24.2	28.2	10.6	-	-
SV 2	45	4.36	31.8	12.24	11.1	21.1	3.29
SV 3	67	3.82	26.4	22.2	9.6	11.9	3.00
SV 4	35	3.92	31.4	10.16	10.5	22.7	3.53
SV 5	80	3.5	19.6	28.1	10.8	17.5	3.41
UV 1	40.5	4.17	25.5	15.2	11	15.9	6.89
UV 2	45	4.52	27.4	13.6	10.6	19.1	6.76
UV 3	51	3.59	16.8	15.8	10.6	11.7	4.19

### 2.3 Calculation of mechanical properties index

Assuming an open section of the vessel as a rectangle, its cross section area is determined by multiplying vessel’s perimeter to wall thickness. L_0_ stands for primary length of the vessel, l_0_ (mm) is the perimeter of the vessel and S_0_ (mm^2^) is its primary area. Engineering stress is then calculated by:

(2) $$\sigma_{\mathrm{eng}}=\frac{F}{S_{0}}$$

Where $\sigma_{\mathrm{eng}}\left(N/mm^{2}\right)$ and F(N) indicate engineering stress and exerted force correspondingly. Blood vessel is assumed to be incompressible, thus, blood vessel’s instant area is S=S0L0L. Cauchy stress is also determined as:

(3) $$\text{σ}=\frac{F}{S}$$

Where $\sigma \left(N/mm^{2}\right)$ indicates instant stress or Cauchy stress, so we can have the following equation:

(4) $$\sigma =\lambda \sigma_{\mathrm{eng}}$$

Most of the biological soft tissues have nonlinear viscoelastic rheological behavior, so a nonlinear anisotropic viscoelastic model, as introduced by Fung in 1972 [26], can be developed to show the nonlinear behavior of the tissues. The best fit curve showing the nonlinear behavior of our results was a fourth order polynomial.

(5) $$\text{σ}\left(\lambda \right)=a\lambda^{4}+b\lambda^{3}+c\lambda^{2}+d\lambda +e$$

Where *a, b, c, d* and *e*$\left(N/mm^{2}\right)$ are constants. Then, instant elastic moduli $\left(E\left(N/mm^{2}\right)\right)$ of soft tissue could be determined as the slope of stress-stretch graph as follows:

(6) $$\text{E}=4{\text{aλ}}^{3}+3{\text{bλ}}^{2}+2\text{cλ}+\text{d}$$

## Results

Apparatus output is in volts and computer changes these data to displacement and force. Obtained data was imported in MATLAB (Mathworks, MA, USA 2010) and in order to reduce any probable noise, the average of 3 data points was estimated for each point. Finally, to obtain Cauchy stress and stretch ratio, equations 1–4 were utilized. The fixation method applying paper tissue and sand paper was pretty suitable and samples were completely fresh without any destruction in the tissues macroscopically.

### Histology

To investigate and analyze final mechanical results and to find a better and more precise perspective on the conducted research work, morphology of the samples was also investigated. The staining procedure took 24–36 hours to complete and microstructural components as well as their proportion were evaluated semi-quantitatively with the help of a pathologist. Wall thickness of HUV was significantly greater than that of SV (1.48 (mm) versus 0.51(mm) on average) while in contrast, lumen diameter in HUV was smaller than SV.

**Figure 2:**
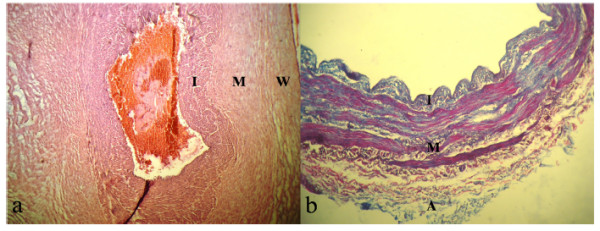
**Histological section of HUV and SV.** The wall may be divided into Intima (I), Media (M), and Adventitia (A) or Wharton jelly (W). **a**) Haemotoxylin and Eosin (HE) stained section of HUV. Layers M and W are not uniform (magnification × 100) but thicker than that of SV. **b**) Van Gison’s stained section of SV. Collagen fibers are blued leafs while red strings stand for smooth muscles. Adventitia is mainly composed of collagen fibers (magnification × 400).

Three different layers of intima, media, and adventitia (in case of HUV it was Wharton jelly) were easily detected especially in case of HUV, where components’ orientation and density changed considerably in different layers. In intima, endothelium cells as well as subendothelial layer and inner elastic lamina were observed and collagen fibers content of HUV was sparse. In media, which was the thickest layer of SV, but not of HUV, the greatest area was occupied by smooth muscle cells in either specimen, while collagen fibers could be detected hardly in the latter case. For HUV, this layer did not enjoy a uniform thickness, however, the main difference between SV and HUV was observed in adventitia layer, where collagen content of SV, with higher interwoven density, was far greater than that of HUV. Also, from geometrical point of view, Wharton jelly, again with a non-uniform thickness, was thicker than adventia of SV (Figure 2).

### Stress-stretch diagram of both vessels

Elastin fibers are the first engaged elements enduring the tension and as cross head goes farther, collagen fibers are gradually recruiting in tension [2,18]. This coincides with a non-linear escalation in stiffness of blood vessel and further tension ends in the rupture of elastin fibers at first. Applying further tension results in tearing of collagen fibers that is synchronal with a collapse in the stiffness of the artery.

**Figure 3:**
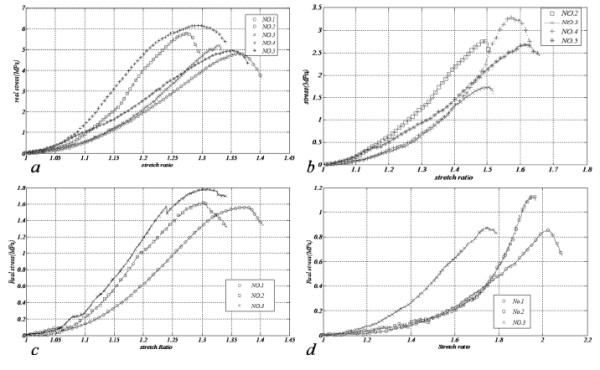
Real stress versus stretch ratio, a) longitudinal SV, b) circumferential SV, c) longitudinal HUV, d) circumferential HUV.

Figure 3 (a-d) shows real stress versus stretch ratio for both vessels in either direction. With marginal noises, all of the samples demonstrated a non-linear behavior demonstrating the necessity of study of these tissues in both longitudinal and transversal directions. Slope of each graph is zero at the beginning escalating markedly in higher stretch ratios and then ultimate strength of the tissue is recognizable by a sudden fall in stress-stretch curve. Because of noticeable stretch ratios and based on equation 4, if engineering stress is used to determine the stress of the vessel, there would be a tremendous difference between Cauchy stress and engineering one. As veins are lengthened, cross section of which diminishes leading to greater stresses in the blood vessel. Collagen fibers are the strongest elements of the vessel, thus, they are the last elements to tolerate tension. Table 3 depicts the ultimate strength and its corresponding stretch ratio for each vessel in both directions.

**Table 3:** Ultimate strength and corresponding stretch ratio

**Sample**	**1**		**2**		**3**		**4**		**5**	
SV- Longitudinal	σ (MPa)	*λ*_*m*_	σ (MPa)	*λ*_*m*_	σ (MPa)	*λ*_*m*_	σ (MPa)	*λ*_*m*_	σ (MPa)	*λ*_*m*_
SV- Circumferential	4.81	1.37	5.74	1.27	5.20	1.33	6.23	1.29	4.92	1.35
UV- Longitudinal	-	-	2.78	1.49	1.69	1.5	3.28	1.57	2.69	1.62
UV- Circumferential	1.58	1.37	1.61	1.30	1.78	1.31	-	-	-	-
SV- Longitudinal	σ (MPa)	*λ*_*m*_	σ (MPa)	*λ*_*m*_	0.87	1.74	-	-	-	-

An acceptable correlation was found between results of each test group, making the recognition of vessels strength and their stiffness uncomplicated. SV describes higher non-linearity with regard to HUV and while the collapse in longitudinal specimens was abrupt, circumferential samples tore with a flatter pattern which was detectible during the tests. With 6.23 (MPa), SV in longitudinal tension enjoyed the greatest strength while circumferential UV samples demonstrated the least and in terms of stretchability in longitudinal tension, both of the vessels showed comparable results, even though UV extended far more than SV in circumferential tests.

In order to shed light on the results, average stiffness and stretch ratio as well as their standard deviations (STD) have been shown in Table 4. This way, comparing the results would be easier.

**Table 4:** Average and standard deviation for strength and stretch ratio of both vessels

	**σ (MPa)**	***STD***	_***λ***_	***STD***
SV- Longitudinal	5.38	0.6	1.32	0.04
SV- Circumferential	2.61	0.67	1.55	0.06
UV- Longitudinal	1.65	0.11	1.33	0.04
UV- Circumferential	0.97	0.19	1.90	0.16

On average, stiffness of the SV in both directions is obviously greater than that of HUV. While SV endures longitudinal stress up to an average of 5.38(MPa), HUV can only tolerate 1.65(MPa) of longitudinal stress. This is a 225% increase in longitudinal direction, whereas stretch ratio is virtually similar.

**Figure 4:**
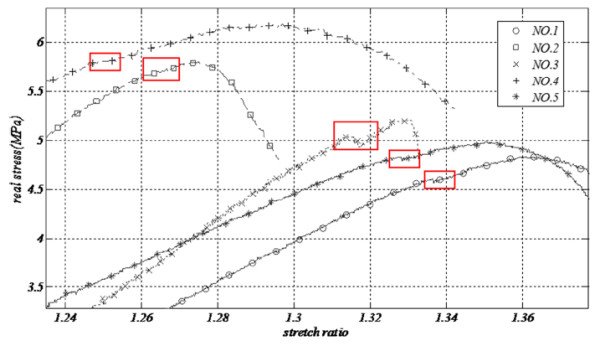
Strength reduction as a result of elastin rupture before specimen’s rupture near maximum stiffness (SV, longitudinal test).

Considering longitudinal tension, there is a small reduction in the strength of samples that is probably related to elastin rupture. As previously explained, elastin fibers tear before collagens. In longitudinal Saphenous samples shown in Figure 4, this is pretty recognizable, since there is a small collapse and then a bounce back in enduring-stress vessels before ultimate strength of each specimen that is highlighted with a red rectangle in this figure. In circumferential test, however, there was no sensible result to be a sign of rupture of a group of elements ahead of others.

### Estimation of blood vessel’s stiffness with a mathematical model

**Figure 5:**
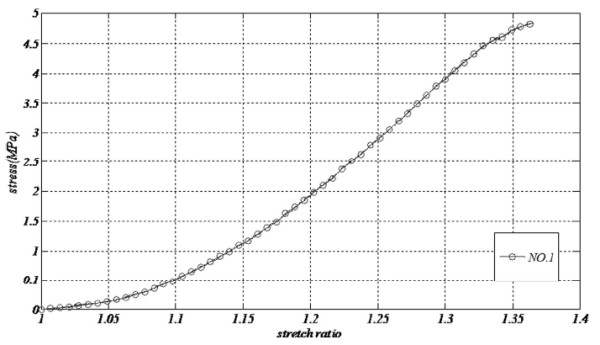
Real stress versus stretch ratio up to maximum strength for longitudinal Saphenous No.1.

**Figure 6:**
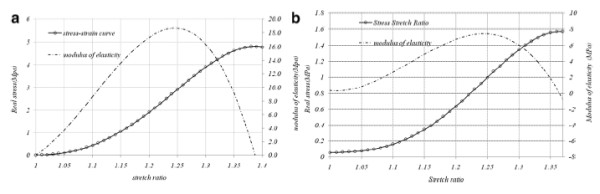
Fourth order polynomial and its derivation for a) SV, b) HUV, both in longitudinal direction.

A model for nonlinear anisotropic viscoelastic tissue, based on what Fung introduced in 1972, was applied to define the non-linear rheological behavior of these tissues [26] and his model was widely employed by other investigators [2]. To obtain the appropriate function to explain the behavior of these vessels, stress stretch formulation of each vessel was assumed to be the maximum stress; take Figure 5 (related to SV No.1 in longitudinal orientation) as an example which is drawn up to λ=1.37. Other polynomials were examined and the best was a fourth order that precisely followed the pattern of $\sigma =f\left(\lambda \right)$. The results were fitted in MATLAB (Mathworks, MA, USA 2010). Figure 6 depicts the fitted result for both HUV and SV with modulus of elasticity shown with a dash-line being scaled in the right hand of each chart. Results of the polynomial had a strong correlation to experimental outputs as the least-square (R^2^) for all fitted diagrams was greater than 0.999.

Instant elasticity of each vessel was calculated by derivation of equation 5. Based on derived equation, in Figure 6 blood vessel’s elasticity and stress-stretch results are respectively shown with dash-line and bullets (the left figure related to SV and the right one is for UV). Launched at zero, strength and modulus of elasticity raise with escalation of stretch ratio for both specimens. Corresponding results show that maximum elasticity of the blood vessel has occurred before the maximum stiffness and considering elastin rupture, which was explained in previous section, this maximum occurs before rupture of these fibers; e.g. in Saphenous No.1, maximum elasticity comes about λ=1.24 while maximum stiffness happens at λ=1.37. In this figure, maximum elasticity for SV and HUV were 18.7(MPa) and only 7.85(MPa) respectively. Table 5 illustrates the respective coefficients of each vessel.

**Table 5:** average and standard deviation for strength and stretch ratio of both vessels

**Coefficient**	***a***	***b***	***c***	***d***	***e***
SV- Longitudinal	−4.4505E8	1.9586E9	−3.1759E9	2.2561E9	−5.9376E8
SV- Circumferential	−7.5200E7	3.4760E8	−5.8460E8	4.2740E8	−1.1520E8
UV- Longitudinal	−2.7456E8	1.2378E9	−2.0699E9	1.5249E9	4.1823E8
UV- Circumferential	−2.5880E5	1.8300E6	−3.5930E6	2.6670E6	−6.3520E5

The contribution of early recruited fibers in the blood vessel can be evaluated by *d,* since it is the slope of the graph when λ→1 and *e* is constant which shifts the graph upward and downward. Greater coefficient in either orientation demonstrates a higher stiffness. Longitudinal SV has the greatest coefficients whereas circumferential HUV has the lowest and since in SV, *d* is in excess of HUV, elastin fibers amounts are higher in the former playing the greatest role in longitudinal SV.

## Discussion

Limitation of autograft blood vessel sources makes application of other alternatives like synthetic sources and allografts inevitable [21]. In terms of long-term patency of these grafts, previous studies have reported elasticity and compliance mismatch between host artery and graft as one of the most imperative reasons of Intimal Hyperplasia (IH), hemodynamic alteration and even graft failure [20]. Therefore, C/E ratio is being introduced as a sensible criterion and effective index that can reflect elastic characteristics and elasticity of the vessel [20]. As C/E increases, stratchability decreases [20].

The present study aimed to determine mechanical properties of human SV and HUV. Blood vessel’s real stress versus stretch ratio was obtained and since it was non-linear, Hook’s law was not an applicable formulation for it. So, a fourth order polynomial was applied to show rheological behavior of these soft tissues. Most of the studies in the literature are conducted based on the uniaxial tensions [2,13-15], nonetheless, some other research works, like that of Lally et al., includes equibiaxial tension as well [27]. The assumption of the study based on uniaxial tension may lead to different results compared to in vivo situation, but based on aforementioned studies, blood vessel has anisothropic mechanical characteristics. Thus, all of the experiments should be performed in both circumferential and longitudinal directions.

Histological investigations, with similar outcomes to previous studies, revealed that HUV comprises less collagen and elastin content with smaller lumen area and thicker wall and higher content of these fibers expresses greater stiffness [18,20,25]. Generally, elastic tissue components decline in the blood vessel in the periphery direction as blood pressure is regulated [25]. Smooth muscles are oriented circumferentially and seemingly do play an important role in circumferential tension [2]. In case of HUV, however, external layer is covered with Wharton jelly to reinforce the vein rather than highly-collagen included adventitia layer [24]. Considering histological aspects, previous studies reported the suitability of HUV for small caliber arteries proposing that this vessel enjoys suitable microstructural components to be applied in a bypass surgery in the absence of Saphenous vein [20].

Both SV and HUV are stiffer in longitudinal direction than that of circumferential: this is because of the great impact of collagen and elastin fibers orientation and their density within the vessels [2,10,18]. Components that constitute blood vessels enjoy dissimilar mechanical properties so that collagen fibers stiffness is greater than elastin stiffness and modulus of elasticity increases as a result of further recruitment of collagens in higher stretch ratios [2,18]. In the blood vessels, they are directed more longitudinally than circumferentially [2,10] and Figure 2 whereas in other tissues such as knee cartilage, they are distributed in all directions [18]. SV is far stiffer than HUV which may be caused by greater content of collagen in SV than HUV, where aforementioned difference even exists in circumferential orientation.

On the same wavelength of vessel’s stiffness, due to the fact that modulus of elasticity is directly proportional to stiffness, this parameter follows similar outcome to stiffness which has been discussed previously. In the case of circumferential HUV, not only stiffness is lower than SV, but also higher stretch ratios lead to noticeable lower elastic moduli.

Stretch ratio for both of the specimens was the same in longitudinal direction that probably shows the stretchability of collagen fibers. As mentioned before, collagens are the last elements to endure tension and as they tear, there is no abiding element anymore. However, in the circumferential direction, mechanism of endurance seems to be a little bit different; here, smooth muscles are beside fibers causing greater stretchability in the circumferential direction, so that HUV can stretch up to an average of 190% which is a 90% increase in circumferential length. On the same way, Miyamotto compared the stiffness of bovine pericardium and SV and found similar results in his studies for both directions [10]. Balaz found similar results for porcine coronary, although these vessels were more elastic than human coronary [14]. Furthermore, some researchers like Rossmann could simply distinguish two regions in which just elastin fibers were engaged and the area in which collagen was taking part beside elastin fibers [13].

Donavan’s investigations on SV showed 24.24 ± 6.67 (N) for ultimate strength with 8% difference concerning our studies with 26.67 ± 5.11 (N), but wall thickness was 0.38 (mm) in his studies; while our specimens had 0.51 ± 0.03 (mm) on average and this may be as a result of the fact that our Saphenous samples were harvested from thigh but in his study samples were from both thigh and leg [9]. Miyamotto et al. reported 4.9 (MPa) as maximum strength of SV, which is 9% lower than our outputs with an average strength of 5.38 ± 0.6 (MPa) [10].

Focusing on longitudinal tension of SV, a small collapse is observed in graphs. Balaz explained this phenomenon to be a sign of rupture of other extra components like lipid clots, however, in our experiments, specimens were clearly stripped and washed from extra tissues and this may not be as a result of slipping, since it came about in all samples which were tightly fastened to the jaws. Seemingly, elastin rupture is the main cause of this decrease because similar results for elastin rupture were recognized in SVs, with Miyamotto [10].

Mathematical model of a soft tissue has rarely been described by applying curve fitting [Assoul]. Curve fitting on the stress-stretch results of these veins gave us a better sight and the possibility of a clearer comparison between these two veins. These coefficients were greater in the SV showing that SV is stiffer than HUV. The applied method can be established as one of the standard methods for all types of soft tissues and the coefficients of these equations can be listed in different tables in terms of various parameters. Thereby, comparison between two different vessels is easily possible and can be used as a quick reference. Consider the Assoul’s study on aortic vessels of rat as an example; these coefficients are depicted in Table 6 for thoracic aorta. They are lower than those of SV, but a little greater than HUV demonstrating the probability of the greater density of collagen and elastin fibers in the aorta of rat [2].

**Table 6:** **Coefficient of fourth order polynomial for thoracic aorta**[2]

	***a***	***b***	***c***
longitudinal	−4.08E7	8.82E7	−4.42E7
circumferential	−4.88E7	10.62E7	−5.72E7

Measuring of the samples, there were local anatomical changes in wall thickness, but we assumed samples to be like a uniform pipe and this may have slightly affected the results. Although the number of truly-tested samples was not enough, the present research work represents an acceptable view of the stiffness of HUV. Initial length of specimens were also measured at 0.1(N) and this may slightly affect the stress and stretch ratio for different vessels.

Referring to many studies which have focused on the effect of elongation rate, applied rate of elongation in current study was quite low (1(%/s)). For instance, Assoul had a comprehensive scrutiny on the rate of frequency (1–7.5 (Hz)) in cyclic tension and rate of elongation (1–10 (%/s)) in direct tension for rat thoracic aorta reporting no correlation between rate of elongation and elastic moduli [2]. Rossman studied bovines’ veins at higher rates of elongation (up to 100%/s) revealing that only ultimate strength is slightly affected by cross head pace [13], therefore in conformity with the previous literature, it is quite predictable that our rate of elongation did not change the results significantly.

To sum up, higher stretches in grafts in addition to cyclic loads boosts the hypothesis that aneurysm or even rupture in the less stiff vessels may take place sooner, especially when considering high, pulsatile pressure in the aorta. Before harvest, HUV experiences lower blood pressure than SV and higher blood pressure leads to greater amounts of fibers, and consequently greater stiffness. Thus, as a result of low stiffness in this vessel in comparison with SV, if we use this vessel for bypass operation, there would be higher chances of bypass aneurysm by the time as previous experiences have shown [20,21,25]. Additionally, non-uniform thickness in HUV may result in a faster aneurysm or even rupture, so we can deduce that, although using this vessel is more prevalent than synthetic grafts, and especially in femoral cases, it is not a decent choice for aortic bypass surgery.

To make vessels stiffer, chemical intervention or physical reinforcement such as wrapping and tissue engineering, as an emerging field, is widely applied nowadays that can be our next step in development of the decent graft.

## Conclusion

The main purpose of this study was to further investigate and to compare two blood vessels’ stiffness and appraise the feasibility of HUV as a detour in CABG. In this study, we categorized three main methods for determination of elastomechanical properties of blood vessels and universal testing machine is used for obtaining stress-stretch results. Due to large deformation problems, engineering relations for stress and stretch were found to be inappropriate and then Cauchy stress–strain formulation was introduced.

HUV’s stiffness was found to be much lower than those of SV making it indecent for CABG purpose. Apparently, one reason that makes the mammary artery a better vessel for CABG is its stiffness that leads to a better long-term patency.

It was also observed that HUV does not enjoy a uniform thickness along its cross section which must be taken into account. Thus, its lower stiffness as well as inhomogeneous thickness are two main downsides of HUV and because of high pulsatile pressure in the coronary, it is a susceptible vessel to early aneurysm or even rupture in CABG.

## Competing interests

The authors declare that they have no competing interests.

## Authors’ contributions

The protocol of test was prepared with BAH and HAT. Tests were performed with BAH and mathematical models were obtained with MN and BAH. All of the members participated in analyzing the results. Writing and modification of article was all of the authors’ responsibility. All authors read and approved the final manuscript.
